# Innate Responses Induced by Whole Inactivated Virus or Subunit Influenza Vaccines in Cultured Dendritic Cells Correlate with Immune Responses *In Vivo*

**DOI:** 10.1371/journal.pone.0125228

**Published:** 2015-05-01

**Authors:** Maaike Stoel, Judith Pool, Jacqueline de Vries-Idema, Fatiha Zaaraoui-Boutahar, Maarten Bijl, Arno C. Andeweg, Jan Wilschut, Anke Huckriede

**Affiliations:** 1 Department of Medical Microbiology, University of Groningen, University Medical Centre Groningen, Groningen, The Netherlands; 2 Department of Viroscience, Erasmus University Rotterdam, Rotterdam, The Netherlands; The University of Adelaide, AUSTRALIA

## Abstract

Vaccine development involves time-consuming and expensive evaluation of candidate vaccines in animal models. As mediators of both innate and adaptive immune responses dendritic cells (DCs) are considered to be highly important for vaccine performance. Here we evaluated how far the response of DCs to a vaccine *in vitro* is in line with the immune response the vaccine evokes *in vivo*. To this end, we investigated the response of murine bone marrow-derived DCs to whole inactivated virus (WIV) and subunit (SU) influenza vaccine preparations. These vaccine preparations were chosen because they differ in the immune response they evoke in mice with WIV being superior to SU vaccine through induction of higher virus-neutralizing antibody titers and a more favorable Th1-skewed response phenotype. Stimulation of DCs with WIV, but not SU vaccine, resulted in a cytokine response that was comparable to that of DCs stimulated with live virus. Similarly, the gene expression profiles of DCs treated with WIV or live virus were similar and differed from that of SU vaccine-treated DCs. More specifically, exposure of DCs to WIV resulted in differential expression of genes in known antiviral pathways, whereas SU vaccine did not. The stronger antiviral and more Th1-related response of DCs to WIV as compared to SU vaccine correlates well with the superior immune response found in mice. These results indicate that *in vitro* stimulation of DCs with novel vaccine candidates combined with the assessment of multiple parameters, including gene signatures, may be a valuable tool for the selection of vaccine candidates.

## Introduction

Vaccination is the cornerstone in the control of many infectious diseases. The incidence of infections like tetanus, measles, rubella, and polio, has declined dramatically after the introduction of childhood vaccination against the pathogens causing these diseases ([1,2]). Nevertheless, there is a great need for novel and improved vaccines. No vaccines are available yet for viruses such as HIV and Dengue virus (reviewed in [3]). In addition, there are many vaccines that only confer a low level of protection, the *Mycobacterium bovis* bacillus CalmetteGuérin (BCG) vaccine against tuberculosis being a good example [4]. A better knowledge of the mechanisms involved in immune response induction by vaccines could greatly facilitate vaccine development.

Dendritic cells (DCs) are known to play a central role in both the innate and the adaptive immune response against infectious organisms. Binding of microbes or microbe components to pattern recognition receptors (PRRs) expressed by DCs, leads to the activation of various signaling pathways within these DCs, resulting in the expression and secretion of co-stimulatory molecules, chemokines and cytokines [5,6]. These DC-derived effector molecules regulate the recruitment and activation of cells from the immune system and ultimately determine the magnitude and phenotype of the resulting adaptive immune response. The same mechanisms also apply to vaccine-evoked immune responses. We, therefore, studied whether distinct responses of DCs to different vaccine formulations could be identified *in vitro* and whether these responses correlate with the immune responses these vaccines elicit *in vivo*. To this end we selected two influenza vaccine formulations, whole inactivated virus (WIV) and subunit (SU) vaccine, with well characterized immune response stimulating properties. WIV vaccines are produced by treatment of live influenza virus with β-propiolactone or formaldehyde and retain both the composition and the structure of the native virus. SU vaccines, on the other hand, are produced by solubilization of inactivated virus followed by removal of the viral nucleocapsid and purification of the viral membrane proteins hemagglutinin (HA) and neuraminidase [7]. WIV vaccine has been found to induce superior immune responses over those induced by SU vaccine in mice as well as in unprimed human individuals. In particular, WIV evokes higher hemaglutination inhibition (HAI) titers [8–12], generates a more favorable Th1-type response [8], and induces cross-protective cytotoxic T cells in mice [13]. These differences can be largely attributed to triggering of TLR7 by single-stranded viral RNA which is present in WIV but not in SU vaccines [14].

To evaluate if *in vivo* immunogenicity is reflected by DC reactions *in vitro* we studied the expression of activation markers, secretion of cytokines and the gene expression signature of murine bone-marrow-derived conventional DCs (cDCs) upon stimulation with WIV or SU influenza vaccine or, for reasons of comparison, with live influenza virus. We show that WIV and SU influenza vaccines induce different activation levels as well as distinct gene expression profiles in cultured DCs. These *in vitro* vaccine signatures correlate well with immune responses elicited by these vaccines *in vivo*.

## Material and Methods

### Virus and vaccines

Egg-derived influenza A/Panama/2007/99 (H3N2) virus (A/Pan) and SU vaccine produced from this strain were kindly provided by Solvay Biologicals (Weesp, Netherlands). WIV vaccine was produced by incubation of the active virus (AV) with 0.1% β-propiolactone (Acros Organics, Geel, Belgium) in sodium citrate buffer (125 mM sodiumcitrate, 150 mM sodium chloride, pH 8.2) for 24 hours at RT under continuous stirring. After inactivation, the virus was dialysed against Hepes-buffered saline containing 0.1 mM EDTA (HNE buffer). This inactivation procedure was performed twice. Protein content in AV, WIV and SU was determined by Lowry assay [15]. HA content was assumed to be one third of the total viral protein for WIV (based on the known protein composition of influenza particles and the molecular weight of the viral proteins) and to be equal to the total protein for SU. Equal HA amounts in the vaccine preparations were verified by SDS page. RT-qPCR was performed to determine the RNA content of the vaccines using primers specific for the NP- and the M1-encoding segment. Residual RNA in SU was found to be about 0.5% of the RNA present in AV and in WIV (starting material normalized on basis of HA content).

### Animals

Twenty 16- to 17-week-old specified-pathogen-free female BALB/c mice purchased from Harlan Netherlands B.V. (Zeist, the Netherlands), were used for isolation of bone marrow cells for *in vitro* stimulation experiments. The protocol for this animal experiment was approved by the Animal Experimentation Ethical Committee of the University of Groningen (DEC 4381).

### Culture and activation of DCs

Bone marrow (BM) was flushed from mouse femur using Iscove’s Modified Dulbecco’s Medium (IMDM; Invitrogen, Bleiswijk, Netherlands). BM cells were seeded at 2x10^6^ cells in a 100-mm petri dish (Corning, Amsterdam, Netherlands) in the presence of 200 U/ml recombinant mouse (rm) GM-CSF (Peprotech, London, UK) as described in detail by Lutz *et al*. [16]. After 9 days of culture, the non-adherent cells were collected by gentle pipetting and 5 minutes of centrifugation at 300 g at RT. About 90% of the non-adherent cells expressed the cDC marker CD11c as revealed by staining with PECy5-labeled anti-CD11c (BD Biosciences) and flow cytometry. 1.5x10^7^ cDCs were seeded into a 100 mm tissue culture plastic dish (Corning) in 10 ml fresh medium containing 100 U/ml rmGM-CSF. To induce activation/maturation of the cells, cDCs were exposed to active virus using a multiplicity of infection (MOI) of 1.5, or to different vaccine formulations (10 μg HA per ml). After 4, 12 and 24 hours of incubation at 37°C non-adherent cDCs were harvested for flow-cytometric analysis and RNA extraction, and supernatants were collected for cytokine quantification by multiplex immunoassay.

### Flow cytometry

Characterization of cDCs after exposure to various antigens was performed by flow-cytometric analysis. At the indicated times, cells were double-stained with monoclonal antibodies against CD11c (PECy5) and MHC class II (FITC), CD80 (FITC), CD86 (FITC), CD40 (PE; BD Biosciences), or intracellular influenza NP (FITC; Abcam). Acquisition of the samples was performed on a flow cytometer (FACSCalibur, BD, Breda, Netherlands). A live cell gate was set on the basis of the forward/sideward scatter and the gated cells were further evaluated for surface marker expression. Final analysis was performed using WINlist and WinMDI. 10000 cells were analyzed per sample.

### Cytokine quantification

Cytokine levels were determined using a commercial multiplex cytokine assay (LINCO Research, Inc, Missouri, USA) following the manufacturer’s protocols. Samples were analyzed on a Luminex 100 apparatus, and calculations were performed using STarStation software (Applied Cytometry Systems, Sheffield, UK).

### RNA extraction

Total RNA was isolated using TRIzol (Amersham, Roozendaal, Netherlands) to initially lyse the cells and solubilize nucleic acids and proteins. Then chloroform was added and the watery and organic phases were separated by centrifugation. RNA was purified from the aqueous phase using the RNeasy mini extraction kit (Qiagen, Hilden, Germany). The RNA content was determined spectrophotometrically and the quality was confirmed using the RNA 6000 Nano Assay with the Agilent 2100 Bioanalyzer (both Agilent Technologies, USA). RNA samples were defined to be of sufficiently high quality if the electropherograms generated by the Bioanalyzer demonstrated distinct 18S and 28S ribosomal RNA peaks, and when the RNA Integrity Number (RIN) was above 8.0.

### Microarray analysis

Total RNA (2.5 μg) was used as a template to synthesize double-stranded cDNA and biotin-labeled cRNA, using the GeneChip Expression 3′-Amplification IVT Labeling kit (Affymetrix,USA). Labelling was performed according to the manufacturer's instructions (Affymetrix, USA). Fragmented cRNA was hybridized to mouse Affymetrix 430 2.0 microarrays, using an Affymetrix hybridization Oven 640, washed, and subsequently scanned on a GeneChip Scanner 3000 (Affymetrix).

Expression intensities were log transformed and normalized with the Variance Stabilization normalization method using the R open statistical package. Differential gene expression was assessed by Limma analysis [17]. Probe set wise comparisons between the experimental conditions were performed and correction for multiple testing was achieved by requiring a false discovery rate (FDR) of 0.05, calculated with the Benjamini-Hochberg procedure [17]. Fold change values for probesets with limma analysis were calculated for treated versus time-matched control. The final criterion for inclusion was a >2-fold change (FDR<0.05). The average log-ratio for treatment to control was calculated per probeset. Ratios per gene were calculated by average probesets in Spotfire. Significantly up- or downregulated gene ontologies were identified using the annotation software package DAVID (http://david.abcc.ncifcrf.gov/). Pathway analysis was performed using the MetaCore Analytical suite (GeneGo Inc.). Microarray data are available in the ArrayExpress database (www.ebi.ac.uk/arrayexpress) under accession number E-MTAB-3115.

### Quantitative RT- PCR

To confirm the microarray data, 17 genes were selected for RT-PCR analysis. Selected genes were chosen on the basis of the microarray results such that the entire spectrum of high and low fold up- or downregulation was covered. The same RNA samples were tested in RT-PCR as were used for microarray analysis using the Applied Biosystems StepOne Instrument. The following PCR conditions were used: 10 min 95 °C followed by 40 cycles of 15sec 94°C and 1min 60 °C. SYBRgreen mastermix from Westburg (Leusden, Netherlands) was used, and the primers were used at 0.44μM concentration. Sequences of the primers are given in the S1 Table.

## Results

To evaluate if WIV and SU vaccine preparations could induce a measurable response in DCs *in vitro*, bone-marrow-derived murine DCs were exposed to SU or WIV vaccines or to AV, all derived from A/Pan, for 4, 12 or 24 hours. The virus and vaccine quantities were chosen such that the vast majority of DCs exposed to active virus or WIV turned positive for influenza nucleoprotein (NP) as indicated by a shift of the entire cell population to higher mean fluorescent intensity (MFI) in FACS analysis after staining with anti-NP antibody (S1 Fig). For DCs exposed to AV the MFI increased over time, indicating *de novo* production of NP. As expected, in WIV-exposed DCs the MFI was low and remained constant, while DCs exposed to SU were negative for NP throughout the experiment. Viability of cDCs cultured with the vaccine preparations was similar to that of controls (about 90%). However, viability of cDCs cultured with AV was diminished at all time points (~70% (4h), ~55% (12h) and ~60% (24h)). Only live cells were included in the analysis of surface markers. At the indicated times, cells and supernatants were collected for analysis of surface marker expression by flow cytometry, cytokine production by multiplex cytokine assay and gene expression by microarray.

### cDCs upregulate the expression of activation markers upon stimulation with live influenza virus and vaccine preparations

**Fig 1:**
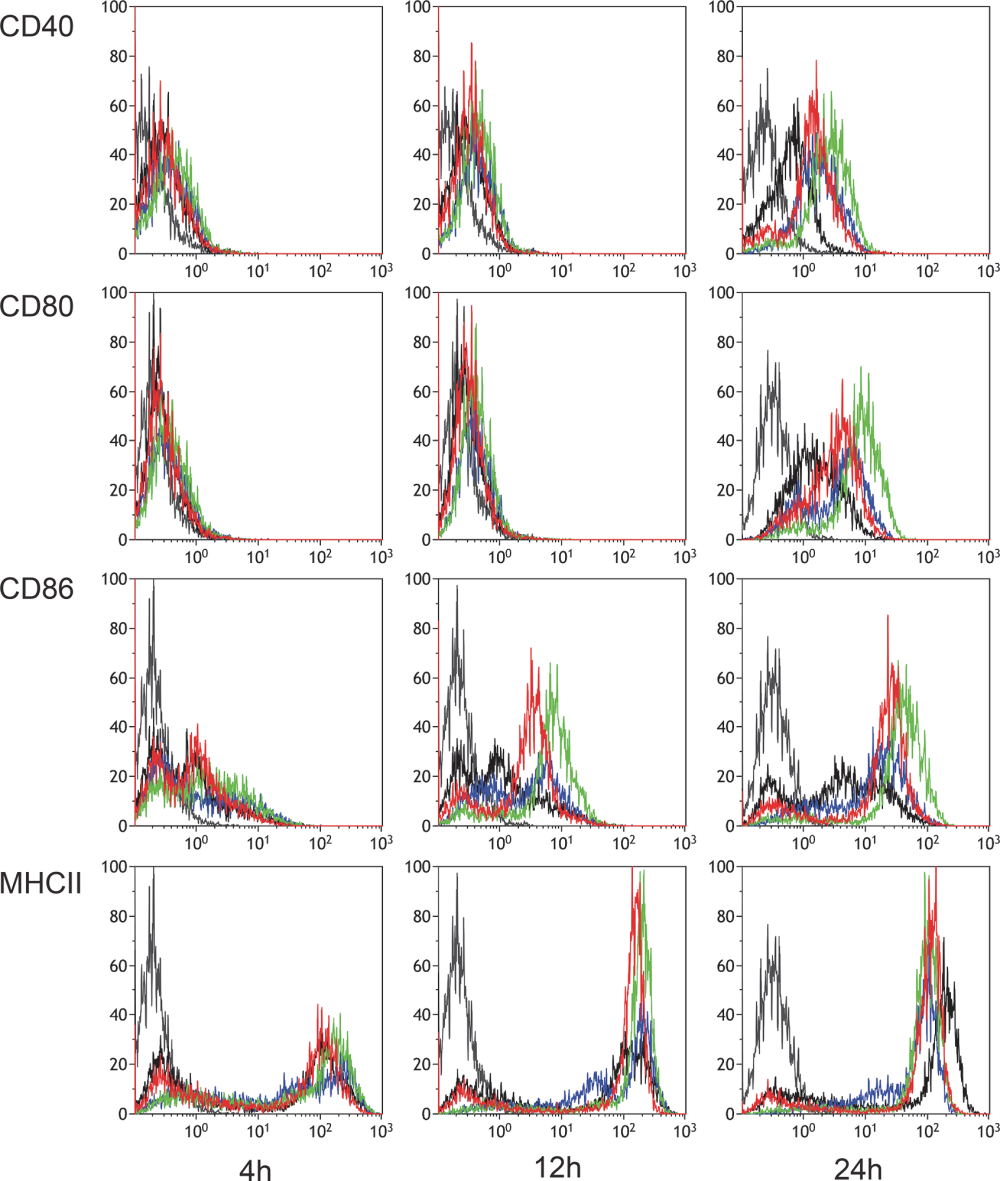
Expression of surface activation markers on murine bone marrow-derived cDCs upon stimulation with virus or vaccine preparations. Bone marrow cells were cultured for 9 days in the presence of GM-CSF. The resulting cDCs were then exposed to active virus (blue), WIV vaccine (green), SU vaccine (red) or medium (black) for 4, 12 or 24 hours. Unstained cells are indicated in grey. Maturation markers CD40, CD80, CD86 and MHCII were analyzed by flow cytometry. Histograms representative for two independent experiments are shown.

Compared to medium-treated controls, DC cultures exposed to either virus or vaccine showed upregulation of the activation markers CD40, CD80, and CD86 over the time course of 24 hours (Fig 1). Expression of CD40 and CD80 was hardly changed after 4 or 12 hours but was significantly increased after 24 hours. With respect to CD86, we could distinguish a marker-high and a marker-low population irrespective of treatment or sampling moment. Upon exposure of the DCs to AV, WIV or SU, the CD86-high population increased both in MFI and in relative proportion over time. WIV induced the most prominent upregulation of each of the three activation markers, exposure to active virus and SU vaccine had a less pronounced and similar effect on CD86 expression. MHC II was expressed to a high level in a proportion of cells from 4 hours onwards. The proportion of MHC II-high cells increased with time in all tested groups, including controls.

In summary, virus and vaccines induced activation of DCs over a time span of 24 hours, as demonstrated by upregulation of the costimulatory molecules CD40, CD80 and CD86.

### WIV but not SU vaccine induces a cytokine response in cDCs comparable to that induced by active virus

**Fig 2:**
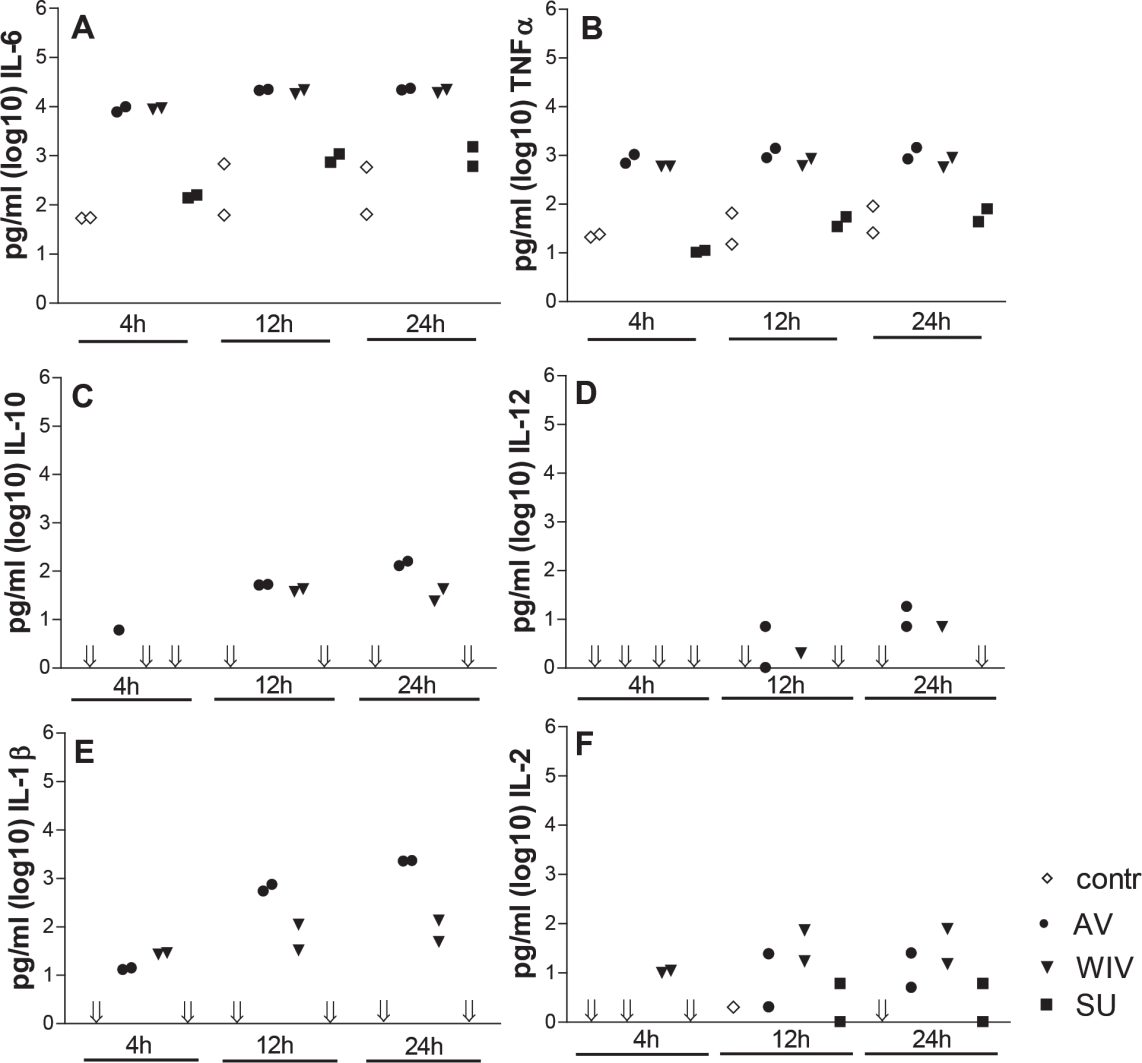
Cytokine production of cDCs upon stimulation with virus or vaccines. Bone marrow cells were cultured for 9 days in the presence of GM-CSF. Cytokine production of these cDCs upon stimulation with active virus (black circles), WIV vaccine (inverted black triangles), SU vaccine (black squares) or medium (open diamonds) for 4, 12 or 24 hours is shown. Supernatants were analyzed for the presence of the indicated cytokines by Luminex technology. The results of two biological replicates for each condition are shown. Arrows indicate values under the detection limit.

As cytokine expression also reveals much about the activation state of the DCs, we measured the cytokine production of virus- or vaccine-exposed DC cultures in a multiplex cytokine assay (Fig 2). Medium-treated control cells expressed detectable amounts of the pro-inflammatory cytokines IL-6 and TNFα but no IL-1β, IL-2, IL-10, or IL-12. DCs treated with SU vaccine, compared to control cells, showed similar levels of IL-6 and TNFα, which increased slightly over time. In addition, SU-treated DCs produced low levels of the T cell mitogen IL-2 at 12 and 24 hours, but none of the other cytokines studied. In contrast, in AV- and WIV-treated DC cultures, IL-6 and TNFα levels were clearly higher than in the control samples throughout the experiment. Already at 4 hours, these cytokines were expressed at maximum level. AV and WIV also induced the secretion of the pro-inflammatory cytokine IL-1β at all times. IL-2 and the Th1-steering cytokine IL-12 were expressed from 12 hours onwards, as was the case for the immune response-dampening cytokine IL-10. The Th2-related cytokines IL-4 and IL-5 were also measured, but expression levels of these cytokines were below the detection limit of the assay. There were only minor differences in expression level of the various cytokines between AV- and WIV-treated cells except for IL-1β which was consistently higher for AV-treated DCs.

In summary, SU vaccine had only minor effects on cytokine expression by DCs, while AV and WIV did induce the secretion of various cytokines and did so to similar extents and with similar kinetics.

### WIV and SU vaccine preparations induce significant modification of gene expression in DCs stimulated *in vitro*

To measure gene expression changes induced by the vaccine preparations or active virus we harvested RNA from DCs at 4, 12, and 24 hours post stimulation and performed Affymetrix mouse 430–2.0 chips assisted genome wide mRNA profiling.

**Fig 3:**
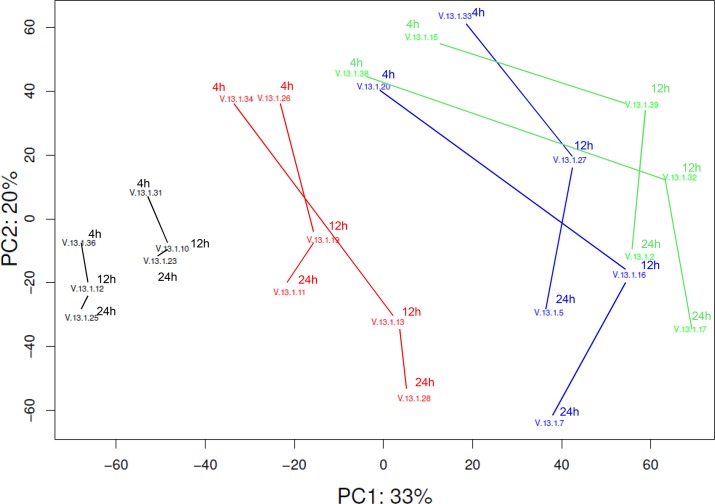
Principal component analysis of stimulation of DCs with AV, WIV, SU or medium. The numbers represent single arrays, with 2 biological replicates per condition tested. The lines have been added to link the samples that have been treated identically but have been harvested at different time points. The medium samples are indicated in black, SU in red, WIV in blue and AV in green. The first and second component explain 33% and 20% of the variation in the data, and correspond to the stimulus used in the experiment and the time after the start of stimulation.

Global gene expression analysis by principal component analysis (PCA) on all microarrays revealed three clusters of samples (Fig 3). The first principal component corresponds with the stimulant used (e.g. medium, AV, SU or WIV), the second principal component corresponds with time since stimulation. The duplicates run for each of the conditions cluster closely. The medium control samples and SU samples cluster separately from each other and from the WIV and AV samples which form a joint cluster.

In a first analysis we aimed to determine the gene expression signatures induced in DCs by AV and the vaccine preparations WIV and SU. To this end, a Limma analysis was performed to identify genes differentially regulated in virus- or vaccine-exposed DCs as compared to the medium control. For analysis only those genes were included that were significantly (FDR<0.05) up- or downregulated compared to the corresponding medium control sample by at least a factor of 2 in at least one of the treatment groups for at least one of the sampling moments. This resulted in the inclusion of a total of 4812 genes for further analyses. The number of genes that were differentially expressed compared to the time-matched medium control at each of the experimental conditions studied is depicted in Table 1. The number of differentially regulated genes was much higher for AV- and WIV-treated DCs than for SU-treated DCs throughout the experiment, with AV-treated DCs showing the largest differences to medium controls. For DCs treated with AV or WIV the number of differentially expressed genes increased up to 12 hours and then remained constant or decreased. Interestingly, at 4 hours mainly upregulation occurred (56–66% of regulated genes), whereas at 12 hours the majority of differentially regulated genes (63–70%) was downregulated. This was also the case at 24 hours although less pronounced (~55% downregulation). For SU-treated DCs relatively minor changes in gene expression were seen which remained more or less constant over the course of the experiment.

**Table 1:** Total number of genes up- or down-regulated in DCs upon stimulation with virus or vaccine preparations.

	Number of differentially regulated genes in experimental condition versus medium-treated DCs		
group	total	up	down
AV 4	569	373	196
WIV 4	869	486	383
SU 4	205	112	93
			
AV 12	2745	812	1933
WIV 12	1595	587	1008
SU 12	125	68	57
			
AV 24	2323	1035	1288
WIV 24	1529	676	853
SU 24	172	55	117

**Fig 4:**
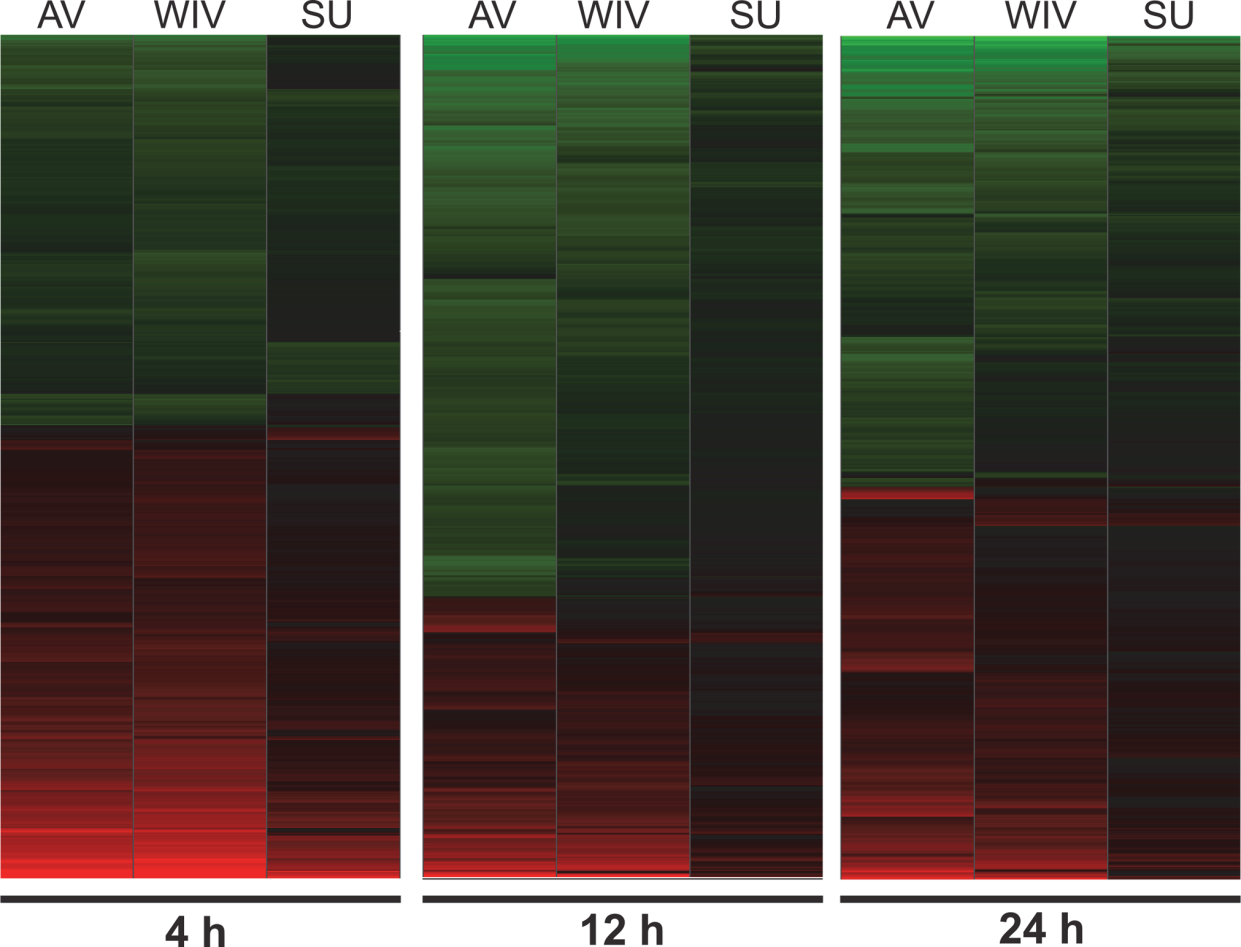
Gene expression of cDCs upon stimulation with virus or vaccine preparations. Murine cDCs were cultured and stimulated as described in the legend to Fig 1. RNA was isolated from the DCs and gene expression was measured by Affymetrix mouse 430 2.0 Genechip array. Two biological replicates per experimental condition were used. Genes were selected that were significantly (p<0.05) regulated compared to the time-matched medium control sample in at least one of the treatment groups at at least one of the sampling moments. This resulted in the inclusion of a total of 4812 genes into further analyses. A heatmap of all differentially regulated genes is shown, where upregulated genes are depicted in red and downregulated genes are depicted in green.

Gene expression was studied and significantly up- or downregulated genes were selected as described in the legend to Figs 1 and 4.

A hierarchical heatmap of all genes significantly up- (red) or down- (green) regulated in at least one treatment group is shown in Fig 4. The expression profiles of AV- and WIV-treated DCs show a high degree of similarity at 4 hours, whereas at 12- and 24 hours the expression patterns start to divert from each other. Noticeably, for SU-treated cells not only the numbers of differentially expressed genes, but also the fold changes in expression level, were overall much lower than for AV- and WIV-treated ones. The pattern of gene expression did not resemble that induced by AV at any time and showed only minor similarities with that induced by WIV.

In order to validate the microarray results the expression of 17 selected genes was measured by qPCR. Selection was done on the basis of the microarray results such that genes covering the entire spectrum of high and low up- or downregulation were included. For all 17 genes, the results of microarray analysis and qPCR correlated well (S2 Table). The Pearson correlation coefficients of the microarray data *versus* the qPCR data were for the 4 hour time point 0.91, 0.93 and 0.70 for AV, WIV and SU stimulated DCs, respectively. For the 12 hour time point, this was 0.86, 0.96 and 0.64, and for the 24 hour time point it was 0.85, 0.69 and 0.81 for the respective experimental conditions.

These results reveal that AV and WIV have a strong and similar effect on gene expression of DCs both in terms of the number and identity of up- and downregulated genes and the fold change in gene expression especially at the 4h time point. As expected, with time AV, which is actively replicating, affects the expression of more genes than WIV vaccine. SU vaccine has only moderate effects on gene expression of DCs and these effects are strongest after short incubation.

### Gene ontology and pathway analyses of regulated genes reveal differences in regulated processes between AV, WIV and SU-treated DCs

To determine which functional groups of genes were regulated in response to the different vaccine preparations, as compared to their medium control, gene ontology analyses were performed using the functional annotation tool DAVID (http://david.abcc.ncifcrf.gov/[18–20]). The top ten hits for each separate condition are listed in S3 Table.

At 4 hours, all top 10 gene ontologies that were upregulated upon stimulation with AV, WIV or SU were related to immune responses (S3 Table). These ontologies included the general immune response ontology and the more specific gene ontologies response to virus, defense response, regulation of cytokine production, innate immune responses, lymphocyte activation and proliferation. The downregulated genes appeared to be involved in RNA processing ontologies (ribosome biogenesis, rRNA processing and metabolic processes and modification) in all three treated groups. At 12 hours, immune response gene ontologies were still upregulated in the AV- and WIV-treated DCs, but no longer in the SU-treated DCs. Downregulated gene ontologies again included RNA processing genes and were found in all three groups of treated DCs. At 24 hours, in the AV- and WIV-treated DCs, but not in the SU-treated DCs, genes involved in programmed cell death were upregulated in addition to the immune response genes. Genes that were downregulated at this time mostly belonged to ontologies involved in cell cycle regulation in all three treated groups of DCs.

**Fig 5:**
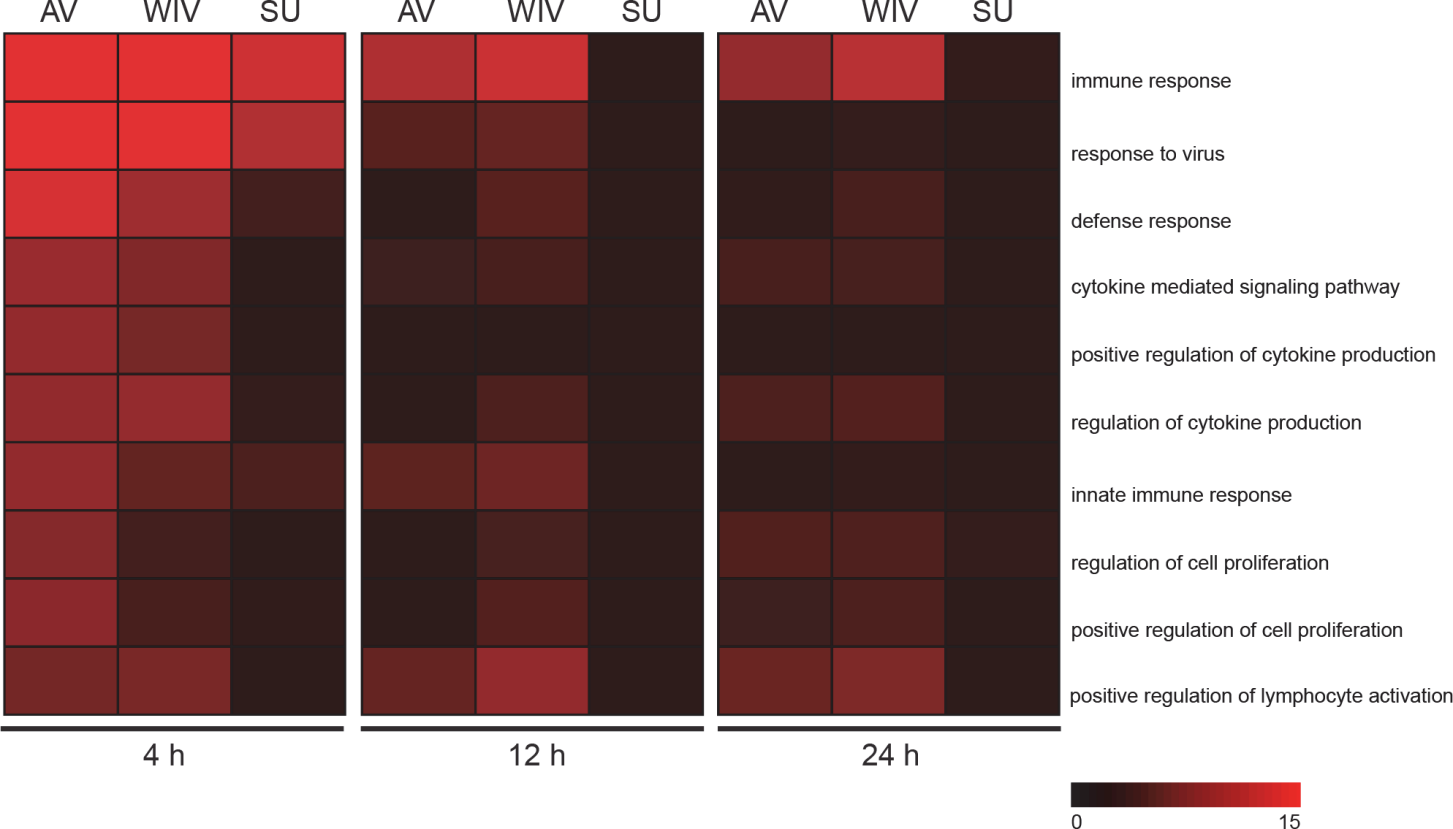
Gene Ontologies (GO) affected in murine bone marrow-derived cDCs upon stimulation with active virus or vaccine preparations. Data sets obtained from the experiment described in the legend to Fig 3 were analyzed for significantly regulated GO using the annotation software package DAVID. The top ten GO identified for AV at t = 4h are listed in the figure. Data are presented in a heatmap. Higher intensity of the red color corresponds to a higher—log p value for the respective GO, thus to a higher probability that the respective GO was affected by the treatment.

The genes within the top 10 immune-related ontologies identified in AV- and WIV-treated DCs at 4 hours were mostly differentially regulated throughout the duration of the experiment, albeit at lower significance levels at the later time points (Fig 5). Fewer genes belonging to the 10 gene ontologies were regulated in DCs stimulated with SU than in DCs stimulated with AV or WIV. Moreover, AV and WIV induced the regulation of genes involved in gene ontologies “immune response” and “positive regulation of lymphocyte activation” for a longer period of time compared to SU.

### WIV vaccine induces a different gene expression signature in DCs than SU vaccine

Previously discussed analysis was aimed at analyzing the gene expression response induced in DCs towards AV, WIV and SU as compared to their time-matched medium controls, which was the first aim of this study. In the following part we will address the second aim of our study, which was to determine if the two vaccine preparations WIV and SU did indeed induce significantly different gene signatures. In order to do so, a direct comparison of WIV- and SU-induced gene expression was performed by Limma analysis. A total number of 515 genes were significantly differentially regulated with a false-discovery rate <0.05 between WIV and SU-treated DCs 4 hours after the start of the stimulation, and this number remained relatively stable at the 12 hour- and at the 24 hour time point (respectively 697 and 540) (Table 2).

**Table 2:** Number of genes differentially regulated in WIV- versus SU-stimulated DCs.

	number of differentially regulated genes of WIV versus SU-treated DCs		
Group	total	up	down
4h	515	285	230
12h	697	361	336
24h	540	409	131

Genes significantly differing in expression in WIV as compared to SU-stimulated DCs were determined by Limma analysis of data from WIV- and SU-stimulated DCs, using an FDR of 0.05.

Gene Ontology analysis using DAVID revealed that genes significantly higher expressed in WIV- compared to SU-stimulated DCs were mainly genes involved in the immune response (S4 Table). More specifically, genes belonging to the gene ontologies antigen processing, response to virus and regulation of leukocyte responses were enriched in the higher expressed genes in the WIV-stimulated DCs. The genes that were expressed at lower levels in WIV- as compared to SU-stimulated DCs did not relate to any significant regulation of specific ontologies (data not shown).

### The phenotype of the gene signature induced by WIV and SU in DCs *in vitro* corresponds with the immune response induced by these vaccines *in vivo*

**Fig 6:**
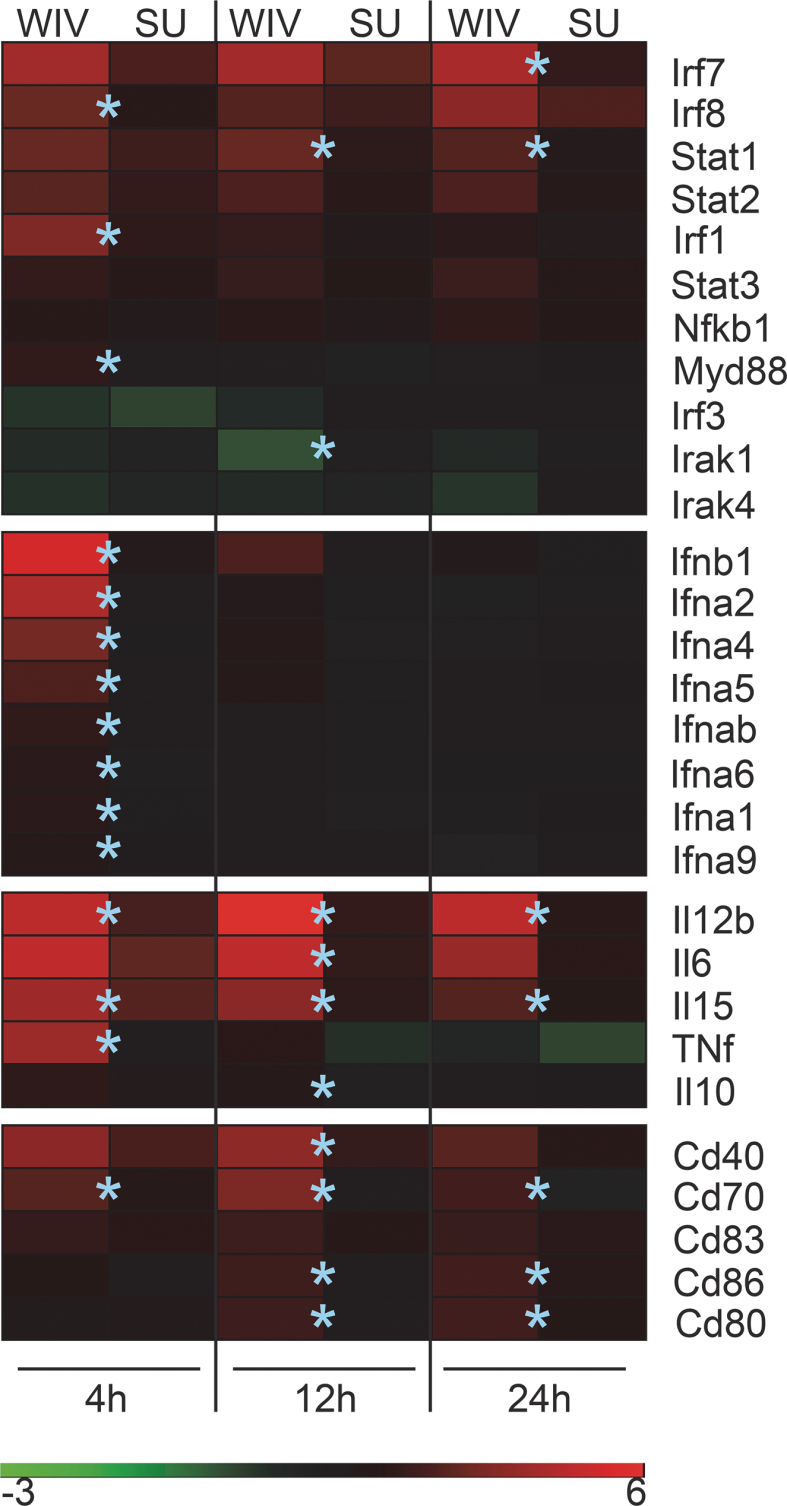
Expression signatures of selected innate immune response-related genes affected by WIV- and SU vaccine. Heatmap showing expression in cDCs stimulated with WIV- or SU vaccine for 4, 12 and 24 hours as compared to time-matched medium control cDCs. A selection of genes is shown that are known to be involved in transcription, the innate- and adaptive immune response. Upregulated genes are shown in red, whereas downregulated genes are shown in green. The asterix (*) indicates that there is a significant difference (p<0.05) in expression between WIV- and SU treated DCs (as measured in a direct Limma contrast between WIV and SU).

We earlier reported that WIV induces type-1 IFN in plasmacytoid DCs and that immunization of mice with WIV results in a strong immune response with a Th1 phenotype. In contrast, SU is not capable of inducing type-1 IFN and immunization with SU results in a moderate response with a Th2 phenotype [8]. To determine if the gene signatures in DCs exposed to WIV and SU correspond with the distinct immune responses these two vaccines induce *in vivo*, we analyzed the gene expression levels of a selection of genes known to be involved in regulating the (Th-balance of the) immune response. The heatmap provided in Fig 6 shows that there were clear differences in the expression of these selected genes between WIV- and SU-stimulated DCs.

Firstly, mRNA encoding for several transcription factors involved in the signaling cascade for the induction of type-1 interferons, such as IRF7, IRF8 and IRF1 were found to be significantly higher expressed in WIV- compared to SU-stimulated DCs. In addition, mRNA encoding for other proteins involved in the type-1 IFN signaling pathways such as MyD88 and STAT1 were also significantly upregulated in WIV- versus SU-stimulated DCs.

Secondly, mRNA encoding for typical effector molecules of the innate immune response, type 1 interferons IFNα and –β, was significantly higher expressed in WIV stimulated DCs at the 4h time point.

Thirdly, genes encoding for cytokines and co-stimulatory molecules that are known to be involved in the humoral and cellular response against influenza virus were more strongly induced in WIV- as compared to SU-stimulated DCs. mRNA coding for the Th1-type response-mediating cytokines IL-12b, the pro-inflammatory cytokines IL-6, IL-15 and IL-1β as well as the co-stimulatory molecules CD40, CD80 and CD86, were expressed to significantly higher levels in WIV- compared to SU- treated DCs, and this upregulation was also maintained for a longer period of time in these cells. The gene expression data for the cytokines- and the co-stimulatory molecules correlated well with respectively the measured cytokine levels (Fig 2) and the expression of co-stimulatory molecules on the surface of these stimulated DCs (Fig 1).

Overall, fine analysis of the microarray data revealed that many immune response genes that are known to have a major effect on the immune response phenotype and magnitude are more strongly induced in WIV- as compared to SU-treated DCs.

## Discussion

In this study, we compared the effects of influenza WIV and SU vaccines, two formulations which largely differ in the immune response they evoke *in vivo*, on cultured murine DCs. The first aim was to elucidate in how far these two vaccine preparations were capable of inducing significant responses in terms of activation, cytokine production and gene expression of the DCs *in vitro*. The second aim was to elucidate in how far the responses of DCs to the vaccines *in vitro* correlate with the differential potency of these vaccines *in vivo*.

Addressing the first aim, we found that both vaccines induced distinct responses in cultured DCs. However, WIV induced higher upregulation of activation markers and increased production of cytokines as compared to SU vaccine. These results are consistent with previous studies by us and others ([8,21]. Moreover, the gene signature induced by WIV profoundly differed from the gene signature induced by SU. More genes were differentially regulated upon stimulation with WIV as compared to SU (when compared to medium-treated DCs) and for genes regulated by both vaccines expression was more vigorously affected by WIV than by SU. Also, WIV activated more signaling pathways associated with immune responses than SU, and activated shared pathways to a higher extent and for a longer time period. These differences in gene signature between WIV- and SU- stimulated DCs are most likely due to the triggering of TLR7 by single-stranded viral RNA which is present in WIV but only in very low amounts in SU vaccines. TLR7 triggering has been shown to result in a strong antiviral immune response, especially the induction of type-1 interferons [22]. The role that TLR7 triggering plays in the superior immunogenicity of WIV versus SU vaccine has been demonstrated before [14]. Theoretically, other RNA-detecting PRRs such as RIG-1 could also be involved in the strong response that was triggered in the DCs by WIV. However, in earlier experiments using Flt3L-mobilized BM-derived DCs we did not find evidence for involvement of PRRs other than TLR7. Rather, when exposing BM-derived DCs from wt and TLR7 k.o. mice to WIV we observed that surface marker upregulation and production of IFNα and IL-12p40 was strictly dependent on TLR7 [14, 22, 23]. On the other hand, involvement of PRRs other than TLR7 might be dependent on the exact cell type studied since we observed that upon stimulation with WIV pDCs purified from the spleens of TLR7 k.o. mice produced almost equal amounts of IFNα as pDCs purified from the spleens of wt mice [14].

Having established that DCs respond to vaccines in a unique manner, the second aim of our study was to elucidate in how far the reaction of DCs to exposure to vaccines *in vitro* reflects the immune response these vaccines elicit *in vivo*. From studies of us and others it is known that in naïve mice but also in naïve humans WIV induces higher HAI and virus-neutralizing antibody titers than split virus (SV) and SU vaccines [8,14,24–26]. In line with this observation, WIV had a much stronger effect than SU vaccine on overall gene expression and on the induction of immune response-related gene ontologies and pathways in cultured DCs. The number of differentially regulated genes and pathways, the extent of up- (and down-)regulation and the duration of differential regulation were all much more pronounced in WIV-exposed than in SU-exposed DCs. Furthermore, the *in vivo* response to WIV in mice resembled that to infection by live influenza virus which is known to be Th1-dominated with large amounts of IgG2a/c and IFNγ-producing T cells [8,24,25,27,28], whereas SV and SU vaccines elicit a Th2 dominated response characterized by IgG1 and Il-4-producing Th cells [8,24,25].

One of the most pronounced differences that we found between the effects of WIV and SU vaccines *in vitro* was the robust induction of mRNA encoding for type-1 interferons and many of its upstream signaling components by WIV, but not, or to a much lower extent, by SU vaccine. Type-1 interferons which are produced *in vivo* immediately upon infection have been associated with the skewing of the immune response towards a Th1-type response by directly interacting with B cells and also T cells [29–33]. Induction of Type-1 interferons is well in line with the Th1 skewing of the immune responses observed *in vivo* upon vaccination with WIV. Consistent with this, we found that WIV-stimulated DCs produced significantly higher amounts of Th1-type response-mediating and pro-inflammatory cytokines like IL-12b, IL-6, IL-1β and TNFα (both mRNA and protein), than SU-stimulated DCs. In particular, IL-12, the key cytokine for skewing of the adaptive immune response to Th1, was found upregulated in WIV- but not in SU-exposed DCs.

We did not detect differences in production of Th2-type response-mediating cytokines like IL-4 and IL-5 between WIV and SU-exposed DCs and indeed production of IL-4 and IL-5 was very low. This is not surprising since the main producers of IL-4 and IL-5 are T cells which were not present in the assay performed. To summarize, quantitative as well as qualitative differences in gene expression in DCs exposed to AV, WIV, or SU matched the Th1 (AV, WIV) and Th2 (SU) phenotype of the immune response evoked by these vaccines *in vivo*.

Although it has to be kept in mind that *in vitro* DC responses might not be predictive for *in vivo* immunogenicity in all cases, several studies have successfully used *in vitro* stimulation of DCs to assess the immunogenic potential of vaccines and adjuvants by determining the capacity of the stimulant to induce the expression of maturation markers and/or cytokine production after 24h of stimulation ([8,20,34,35]). Our study demonstrates that gene expression studies can add to the information obtained from surface marker and cytokine evaluation. Gene expression studies are more sensitive than surface maker and cytokine expression studies. Moreover, gene expression studies allow the simultaneous evaluation of a large number of responding genes and signaling pathways and have thus the potential to provide insight into the working mechanism of a vaccine. Exploitation of this potential needs extended studies with detailed analysis of gene expression patterns combined with verification of the significance of putative response pathways. This was beyond the scope of the current study. Yet, the prospect of defining DC response profiles predictive for vaccine performance *in vivo* is thrilling as such profiles could be very valuable for the selection of promising candidate vaccines as well as for quality control of vaccine batches.

The results described in this study were obtained using murine DCs since that allowed us to directly compare the gene expression patterns induced by exposure of DCs to AV, WIV or SU to the immune responses these agents evoke *in vivo*. As a next step we will elucidate the responses of human PBMCs and/or cultured DCs to the vaccines. Interestingly, gene expression signatures of PBMCs obtained from individuals shortly after vaccination with trivalent inactivated or live attenuated influenza vaccine have recently been shown to have predictive value for the antibody responses at 28 days post vaccination ([36,37]). Future research should establish whether a similar predictive value for *in vitro* stimulated cells (DCs, PBMCs or specific combinations of cells) can be demonstrated and whether *in vitr*o and *in vivo* responses of PBMCs/DCs to vaccines correlate. If so, a systems-biology approach of studying human cells *in vitro* could prove to be valuable for the screening of vaccine candidates.

In summary, we show here that WIV- and SU vaccine preparations elicit distinct immune signatures in cultured DCs *in vitro*, and that, at least for the mouse model, the immune signatures evoked by WIV or SU vaccine *in vitro* correlate well with the magnitude and Th1/Th2 phenotype of the immune responses elicited by these vaccines *in vivo*. Provided that our results in murine DCs can be extended to human cells, we propose that studying multiple facets of the immune response of DCs in a systems-biology like fashion can provide valuable insight into the working mechanism of vaccine candidates. Moreover, studying vaccine/adjuvant candidates on DCs *in vitro* might enable to predict, to a certain extent, immune responses *in vivo* and may therefore serve as an efficient and cost-effective tool for the selection of the most promising vaccine candidates.

## Supporting Information

S1 Fig Intracellular presence of viral NP in murine bone marrow-derived cDCs upon stimulation with virus or vaccine preparations. Bone marrow cells were cultured for 9 days in the presence of GM-CSF. The resulting cDCs were then exposed to live virus (blue), WIV vaccine (green), SU vaccine (red) or medium (black) for 4, 12 or 24 hours. Presence of intracellular NP (visualized by staining with fluorescently labeled antibodies) was analyzed by flow cytometry. Data from one of two independent experiments are shown.
(TIF)

S1 Table qPCR primer sequences for mouse genes. (XLSX)

S2 Table Correlation of gene expression analysis by microarray and qPCR. The relative expression of genes as compared to medium control samples as measured by both qPCR and microarray is presented. The results obtained by microarray and qPCR correlate well.
(XLS)

S3 Table Gene ontologies affected in DCs stimulated with AV, WIV or SU. Genes differentially expressed in DCs stimulated with live virus, WIV or SU vaccine, as compared to medium control were analyzed using the functional annotation tool DAVID. Data is shown for three time points, namely 4,12 and 24h after start of stimulation. Gene ontologies were analyzed using the GOTERM_BP_FAT tool. The top 10 of affected ontologies for both upregulated and downregulated genes is shown. Only those ontologies are shown that are enriched with an FDR of 0.05 or smaller.
(XLS)

S4 Table Gene ontologies significantly higher affected in WIV- than in SU-treated DCs for three different time points. Differentially expressed genes in WIV- versus SU-treated DCs were analyzed using the functional annotation tool DAVID. Gene ontologies were analyzed using the GOTERM_BP_FAT tool. Only those ontologies are shown that are enriched with an FDR of 0.05 or smaller.
(XLSX)
