# Supplementary figures and images for: Innate Responses Induced by Whole Inactivated Virus or Subunit Influenza Vaccines in Cultured Dendritic Cells Correlate with Immune Responses In Vivo
Source: PLoS One. 2015 May 1;10(5):e0125228. doi: 10.1371/journal.pone.0125228 (PMC4416761; doi:10.1371/journal.pone.0125228)

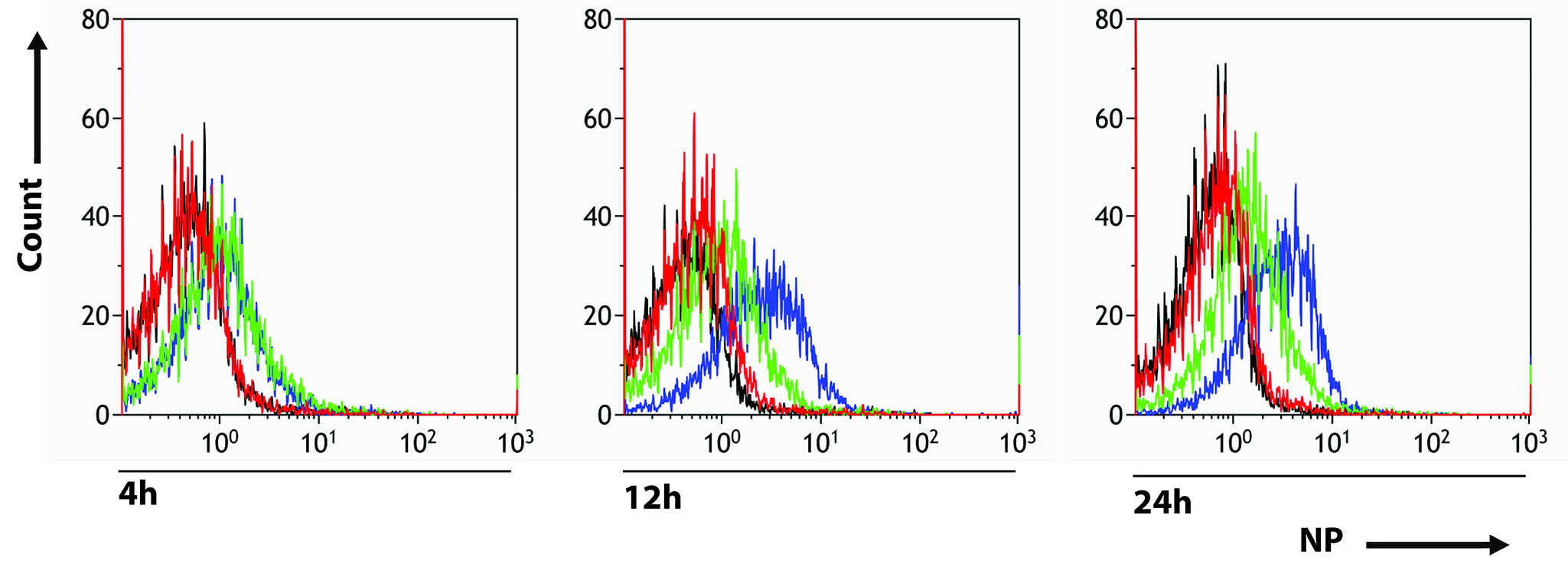

Supplement: S1 Fig — Bone marrow cells were cultured for 9 days in the presence of GM-CSF. The resulting cDCs were then exposed to live virus (blue), WIV vaccine (green), SU vaccine (red) or medium (black) for 4, 12 or 24 hours. Presence of intracellular NP (visualized by staining with fluorescently labeled antibodies) was analyzed by flow cytometry. Data from one of two independent experiments are shown. (TIF) [file pone.0125228.s001.tif]
